# Microbial communication networks governing the ecosystem dynamics of *Salmonella* in poultry environments: ecological interactions, colonization resistance, and emerging control strategies

**DOI:** 10.3389/fbinf.2026.1908527

**Published:** 2026-10-26

**Authors:** M. Naeem

**Affiliations:** Department of Poultry Science, Auburn University, Auburn, AL, United States

**Keywords:** colonization resistance, microbial communication, poultry microbiome, quorum sensing, *Salmonella*

## Abstract

*Salmonella* remains one of the leading foodborne pathogens associated with poultry, posing significant challenges to animal health, food safety, and public health worldwide. Conventional control strategies have largely focused on pathogen elimination through antimicrobials, biosecurity, and vaccination; however, increasing evidence suggests that *Salmonella* persistence is strongly influenced by interactions within complex microbial ecosystems. This review synthesizes current knowledge on how microbial communication networks regulate the colonization, persistence, and transmission of *Salmonella* throughout poultry production systems. Particular emphasis is placed on quorum sensing, metabolic cross-feeding, competitive exclusion, interkingdom signaling, biofilm-associated interactions, and ecological communication occurring within the gut, litter, water systems, and other environmental reservoirs. Recent studies demonstrate that microbial community structure and function influence pathogen behavior through coordinated signaling pathways and indirect ecological interactions that collectively determine colonization resistance and ecosystem resilience. Emerging microbiome engineering approaches, including defined microbial consortia, prebiotics, probiotics, quorum-sensing inhibitors, and ecological network manipulation, offer promising alternatives to conventional pathogen-directed interventions. Integrating microbial ecology with multi-omics technologies and systems biology is expected to facilitate precision microbiome-based strategies for sustainable *Salmonella* control. Viewing *Salmonella* as a participant in interconnected microbial communication networks rather than an isolated pathogen provides a conceptual framework for developing innovative interventions that improve poultry health, enhance food safety, and support One Health objectives.

## 1 Introduction

*Salmonella* remains one of the leading bacterial foodborne pathogens worldwide. Poultry represents one of its most important reservoirs and transmission vehicles to humans (Naeem and Bourassa, 2024; Naeem and Bourassa, 2026; Wang et al., 2025). Despite substantial advances in biosecurity, vaccination, competitive exclusion (CE), feed additives, and antimicrobial interventions, *Salmonella* continues to persist throughout the poultry production continuum, including hatcheries, grow-out farms, processing facilities, and retail products (Velhner et al., 2018; Hameed et al., 2024; Wigley, 2024; Khalefa et al., 2025). This persistent colonization suggests that successful persistence of *Salmonella* depends not only on its intrinsic virulence attributes but also on its ability to interact with and adapt to complex microbial ecosystems (Mahmood et al., 2025).

Conventional research has primarily focused on pathogen virulence mechanisms, host immunity, and direct antimicrobial control strategies (Obe et al., 2023; Naeem and Bourassa, 2024; Naeem and Bourassa, 2026; Fatima et al., 2026). Although these approaches have significantly improved understanding of *Salmonella* pathogenesis, they often consider the pathogen in isolation rather than as an integral component of dynamic microbial communities. Recent detection of antimicrobial-resistant *Salmonella* in poultry manure and slaughterhouse waste further highlights the importance of considering environmental reservoirs within the broader poultry production ecosystem (Kipré et al., 2025). Recent advances in microbial ecology indicate that the poultry gut and associated production environments function as highly interconnected ecosystems, where microbial interactions collectively determine pathogen fitness, colonization resistance, and ecosystem stability (Gart et al., 2016; Kogut and Fernandez Miyakawa, 2023; Nickodem et al., 2025). Consequently, understanding *Salmonella* ecology requires a shift from a pathogen-centered perspective toward a systems-based view of microbial communication networks.

Microbial communication encompasses a broad range of biological interactions through which microorganisms coordinate collective behavior and respond to environmental changes. These interactions include cell-to-cell signaling, metabolite exchange, nutrient competition, cross-feeding, bacteriocin production, biofilm formation, and interkingdom signaling between microbes and the host (Kendall and Sperandio, 2016; Sholpan et al., 2021; Mi et al., 2024). Such communication networks regulate community assembly, ecological succession, stress adaptation, and virulence expression, ultimately shaping *Salmonella*’s ability to establish, persist, and disseminate within poultry ecosystems.

Quorum sensing has emerged as one of the best-characterized forms of bacterial communication. Through diffusible signaling molecules such as autoinducers, bacterial populations coordinate gene expression according to cell density, regulating processes including motility, biofilm formation, environmental persistence, and host invasion (Sholpan et al., 2021; Mi et al., 2024). The relevance of biofilm-associated behavior to poultry environments is further supported by recent evidence demonstrating mono- and dual-species biofilm formation by *Salmonella Typhimurium* and *Staphylococcus aureus* on plastic and galvanized surfaces used in poultry houses (Laban et al., 2024). Equally important are indirect ecological interactions, whereby non-pathogenic microorganisms alter nutrient availability, metabolite composition, or environmental conditions that subsequently influence *Salmonella* behavior. Recent evidence suggests that these indirect interactions may have greater ecological significance than direct antagonism, providing new opportunities for sustainable pathogen control through manipulation of microbial networks rather than elimination of individual pathogens (Jang et al., 2025).

The poultry gastrointestinal tract is a complex microbial ecosystem in which diverse microbial populations interact continuously with host tissues and invading pathogens (Pan and Yu, 2014; Xu et al., 2023). Similar communication networks occur in reused litter, waterline biofilms, hatchery environments, and processing facilities, creating interconnected reservoirs that influence *Salmonella* transmission throughout poultry production (Bucher et al., 2020; Rothrock et al., 2024; Ogundipe et al., 2025). Alterations in these microbial communities affect colonization resistance, biofilm formation, and environmental persistence, demonstrating that *Salmonella* ecology is governed by ecosystem-level interactions rather than individual microbial species (Bottery et al., 2021; Sentenac et al., 2022; Caballero-Flores et al., 2023; Shree et al., 2023).

Studies using defined bacterial consortia and CE cultures further support this ecological paradigm by demonstrating that restoration or engineering of microbial communities can substantially reduce *Salmonella* colonization while simultaneously altering pathogen metabolism and virulence gene expression (Maurer et al., 2024; Naeem and Bourassa, 2024; Wongkuna et al., 2024; Schultz Marcolla et al., 2025). Likewise, manipulation of the gut microbiome through prebiotics or attenuated bacterial strains has been shown to accelerate clearance of *Salmonella* infections by reshaping microbial interactions instead of directly targeting the pathogen (Azcarate-Peril et al., 2018; Naeem and Bourassa, 2024). These findings highlight the importance of viewing microbial communities as functional communication networks that collectively regulate ecosystem resilience and pathogen behavior.

This review examines the role of microbial communication networks in shaping the ecosystem dynamics of *Salmonella* in poultry environments. Particular emphasis is placed on quorum sensing, ecological interactions, CE, host-microbiota-pathogen communication, environmental microbial networks, and emerging ecosystem-based strategies for sustainable control of *Salmonella* within modern poultry production systems. This review was conducted as a narrative review rather than a formal systematic review or meta-analysis; therefore, PRISMA 2020 guidelines were not applied. A conceptual overview of these interconnected communication networks is presented in Figure 1.

**FIGURE 1:**
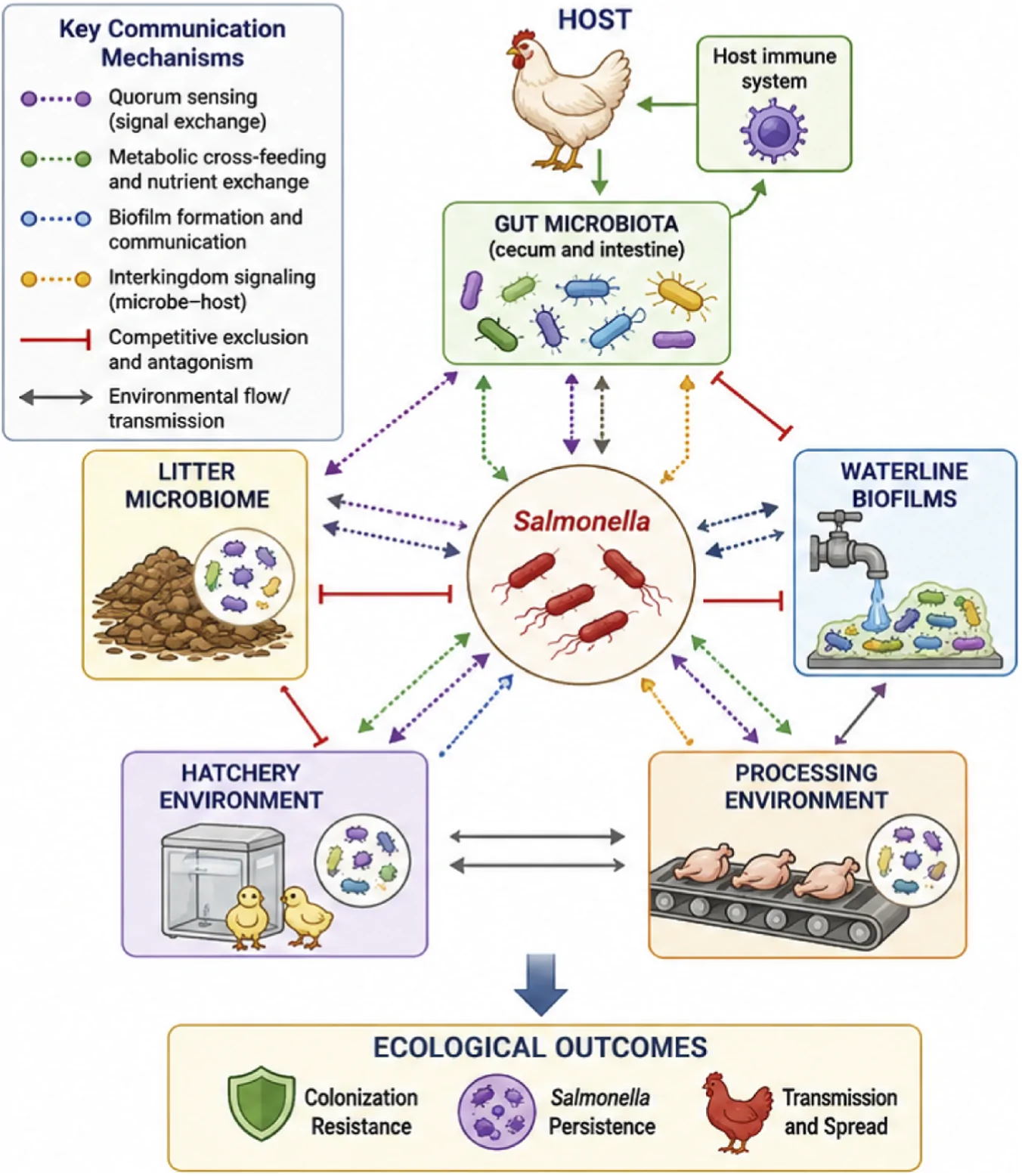
Conceptual overview of microbial communication networks governing *Salmonella* ecology in poultry production. The figure illustrates interactions among the gastrointestinal microbiota, litter, waterline biofilms, hatchery environments, processing surfaces, and host tissues through quorum sensing, metabolic cross-feeding, biofilm formation, interkingdom signaling, and competitive exclusion. These interconnected ecological processes collectively determine colonization resistance, pathogen persistence, and transmission.

## 2 Poultry microbial ecosystems as communication networks

The poultry production system comprises a series of interconnected microbial ecosystems that collectively influence the establishment, persistence, and dissemination of *Salmonella* (Adhikari et al., 2025). Traditionally, the gastrointestinal tract, litter, water distribution systems, hatcheries, and processing environments have been studied as independent ecological niches (Nidaullah et al., 2017; Zamil et al., 2021; Oastler et al., 2022; Shaji et al., 2023; Rothrock et al., 2024; Oladeinde et al., 2025). However, accumulating evidence indicates that these habitats are linked through continuous microbial exchange and communication, creating dynamic networks in which alterations within one ecosystem can influence microbial composition and pathogen behavior in another (Wigley, 2024; Nickodem et al., 2025). Understanding these interconnected microbial networks provides a more comprehensive framework for explaining the ecological success of *Salmonella* and the inconsistent efficacy of many conventional intervention strategies.

The cecum represents the principal ecological niche for *Salmonella* colonization in poultry because of its high microbial density and metabolic diversity (Zeng et al., 2018; Kogut and Fernandez Miyakawa, 2023; Aruwa and Sabiu, 2024; Deng and Wang, 2024). Rather than existing as a passive microbial reservoir, the cecal microbiota functions as an interactive communication network in which microorganisms exchange nutrients, metabolites, signaling molecules, and antimicrobial compounds that collectively regulate ecosystem stability (Braga et al., 2016; Sommer et al., 2017; Douglas, 2020; Jyoti and Dey, 2025; Zhou et al., 2026). Within this complex environment, *Salmonella* must compete for ecological niches while responding to biochemical signals generated by neighboring microorganisms and host tissues (Gart et al., 2016; Kogut and Fernandez Miyakawa, 2023). Consequently, successful colonization depends on ecological compatibility with the resident microbial community rather than pathogen abundance alone.

One of the defining characteristics of resilient microbial ecosystems is colonization resistance, whereby established microbial communities prevent invasion by exogenous pathogens through multiple complementary mechanisms. Although nutrient competition and niche occupation contribute substantially to this phenomenon, recent evidence suggests that microbial communication through metabolic interactions and signaling pathways is equally important (Polansky et al., 2016; Saint-Martin et al., 2024; Gupta et al., 2025; Zhou et al., 2026). Defined bacterial communities isolated from feral chickens significantly reduced *Salmonella* colonization while improving intestinal health, demonstrating that coordinated community function rather than individual bacterial species determines pathogen exclusion (Wongkuna et al., 2024). Similarly, inoculation of broiler chicks with defined microbial consortia increased microbial diversity, reduced Enterobacteriaceae abundance, altered short-chain fatty acid production, and suppressed *Salmonella* colonization, illustrating how ecosystem restructuring can modify pathogen fitness (Schultz Marcolla et al., 2025).

Ecological communication within the gut also occurs through indirect interactions that reshape the metabolic landscape (Gwak and Chang, 2021; Lu et al., 2024; Liang et al., 2025). Microbial populations producing short-chain fatty acids, vitamins, and secondary metabolites influence neighboring organisms without direct physical contact, creating facilitative or inhibitory networks that determine community composition. *Ex vivo* studies comparing permissive and exclusive cecal communities demonstrated profound differences in *Salmonella* metabolism and virulence gene expression, including suppression of SPI-1-associated genes and altered propanediol and vitamin B12 metabolism in restrictive microbial communities (Maurer et al., 2024). These observations indicate that microbial ecosystems regulate pathogen behavior through community-wide metabolic interactions rather than simple antagonism.

More importantly, poultry litter functions as an important environmental communication network connecting successive flocks (Wang et al., 2016; Horyanto et al., 2024; Ekunseitan et al., 2025). Reused litter contains highly diverse microbial populations whose composition changes according to moisture, pH, ammonia concentration, and management practices, generating ecological conditions that influence *Salmonella* persistence. Network analyses have identified specific bacterial taxa associated with reduced *Salmonella* abundance, while *Bacillus subtilis* consortia isolated from litter exhibited inhibitory activity against *Salmonella Heidelberg*, supporting the concept that litter microbiota contribute to natural pathogen suppression through community interactions (Bucher et al., 2020). Rather than serving merely as a contamination source, litter represents an active microbial ecosystem capable of shaping pathogen ecology through ecological succession and microbial competition.

Water distribution systems provide another important microbial habitat in poultry production. Biofilms formed within drinking water lines contain structured microbial communities embedded in extracellular polymeric matrices that facilitate metabolic cooperation, stress tolerance, and cell-to-cell communication. Comparative analyses of litter and waterline biofilms have demonstrated distinct microbial compositions and diversity patterns influenced by water quality history, while the detection of *Salmonella* within mature biofilms highlights their potential role as persistent reservoirs that contribute to flock recolonization despite cleaning procedures (Ogundipe et al., 2025). The structured nature of biofilms promotes close microbial interactions that enhance collective resilience and facilitate long-term environmental survival.

Hatcheries constitute another critical ecological interface where microbial communities established during early life may influence subsequent resistance or susceptibility to *Salmonella* (Rothrock et al., 2024; Sharma et al., 2026). Biomapping studies have revealed considerable serotype diversity within commercial hatcheries, indicating that these environments harbor stable microbial reservoirs capable of introducing pathogens during the earliest stages of production (Rothrock et al., 2024). Early microbial colonization is increasingly recognized as a determinant of community assembly, with pioneer species influencing subsequent microbial succession and ecosystem development. These interactions collectively contribute to colonization resistance by limiting ecological opportunities for invading pathogens.

Environmental persistence of *Salmonella* also depends on communication within multispecies biofilms that are developed on farm equipment, processing surfaces, and environmental substrates. Biofilm-associated microbial communities facilitate nutrient sharing, extracellular signaling, and enhanced stress tolerance while protecting embedded pathogens from disinfectants and environmental stressors. The ability of *Salmonella* to integrate into these structured communities contributes substantially to its persistence within poultry production systems and complicates eradication efforts (Goswami, 2025; Małaszczuk et al., 2025). Consequently, environmental biofilms should be viewed as interactive microbial ecosystems rather than static surface contaminants.

Microbial ecosystems are further characterized by ecological resilience, whereby community function is maintained despite environmental disturbances. This resilience arises from complex interaction networks involving keystone taxa, functional redundancy, and positive or negative feedback loops among microbial populations. Experimental studies have shown that facilitative microbial interaction networks evolve over time and that positive feedback loops often precede major community compositional shifts, suggesting that microbial communication governs ecosystem stability and susceptibility to invasion (Fujita et al., 2023). Such ecological principles may explain why disruption of resident microbiota through stress, dietary changes, or antimicrobial use frequently increases susceptibility to *Salmonella* colonization.

Recent ecological theory proposes that pathogen behavior is frequently regulated by indirect interactions among non-pathogenic microorganisms rather than direct pathogen-pathogen competition. Manipulation of supporting microbial populations can alter pathogen metabolism, virulence, and persistence through cascading ecological effects that propagate throughout the microbial network (Jang et al., 2025). This holistic perspective represents a conceptual departure from reductionist pathogen control strategies by recognizing that ecosystem properties emerge from the collective behavior of interacting with microbial populations.

Collectively, evidence from gastrointestinal, litter, hatchery, and environmental microbiomes demonstrates that poultry production systems function as integrated microbial communication networks in which ecological interactions determine *Salmonella* colonization, persistence, and transmission. Viewing these habitats as interconnected ecosystems rather than isolated compartments provides a mechanistic basis for understanding pathogen ecology and establishes the foundation for ecosystem-based interventions aimed at restoring microbial resilience and enhancing natural colonization resistance. The major microbial ecosystems involved in *Salmonella* communication networks are summarized in Table 1.

**TABLE 1:** Major microbial ecosystems contributing to *Salmonella* persistence and communication within poultry production systems.

Ecosystem	Major communication mechanisms	Ecological role
Cecum	Metabolic cross-feeding, quorum sensing	Colonization resistance
Litter	Community succession, competition	Environmental reservoir
Waterlines	Biofilm signaling	Persistence
Hatchery	Pioneer colonization	Early microbiome establishment
Processing surfaces	Mixed-species biofilms	Cross-contamination

## 3 Microbial communication mechanisms governing *Salmonella* behavior

Microbial processes influencing *Salmonella* behavior within poultry-associated ecosystems can be broadly distinguished according to their mechanism. Dedicated signaling systems, including quorum sensing and interkingdom signaling, involve the production or detection of specific signaling molecules that trigger regulatory responses. In contrast, metabolic cross-feeding and environmental modification influence *Salmonella* indirectly by altering nutrient availability or physicochemical conditions, while extracellular vesicles may mediate transfer of signaling or functional cargo between cells. Horizontal gene transfer represents a related but conceptually distinct evolutionary process involving heritable genetic exchange rather than signal-mediated regulation. Importantly, the strength of evidence for these mechanisms varies considerably. Quorum-sensing and biofilm-associated responses are well characterized in *Salmonella*, whereas the specific contribution of several metabolic, vesicle-mediated, and community-level interactions within poultry-associated *Salmonella* ecosystems remains less clearly established.

### 3.1 Dedicated signaling systems: quorum sensing and interkingdom signaling

Several distinct microbial mechanisms influence *Salmonella* behavior within poultry-associated ecosystems, including quorum sensing, interkingdom signaling, metabolic cross-feeding, biofilm-associated interactions, and environmental modification. These mechanisms differ in how information or ecological effects are transmitted but can interact to influence pathogen fitness, persistence, and colonization (Gart et al., 2016; Kendall and Sperandio, 2016; Sholpan et al., 2021). Among the various communication mechanisms, quorum sensing (QS) is the best characterized and serves as a density-dependent regulatory system through which bacteria coordinate gene expression *via* diffusible signaling molecules known as autoinducers (Hawver et al., 2016; Chu and Yang, 2024). Unlike solitary bacterial responses, quorum sensing allows populations to behave collectively once signaling molecules reach threshold concentrations, synchronizing physiological activities that improve survival under adverse conditions. In *Salmonella*, QS regulates multiple phenotypes associated with environmental persistence and host colonization, including motility, biofilm development, stress resistance, and expression of virulence-associated genes (Sholpan et al., 2021; Mi et al., 2024).

The LuxS-mediated autoinducer-2 (AI-2) system represents the principal microbial communication pathway in *Salmonella* and facilitates communication not only among *Salmonella* cells but also between phylogenetically distinct bacterial species inhabiting the poultry gut and environmental microbiomes (Xavier and Bassler, 2005; Wang et al., 2019; Zhang Q. et al., 2026). Because AI-2 functions as a universal bacterial signaling molecule, it enables *Salmonella* to monitor surrounding microbial populations and adjust its physiology according to community composition (Pereira et al., 2013; Guo et al., 2026). This interspecies communication contributes to ecological adaptation by coordinating nutrient acquisition, metabolic activity, and stress responses within mixed microbial communities (Gart et al., 2016; Sholpan et al., 2021). Rather than acting independently, *Salmonella* continuously interprets signals generated by neighboring microorganisms to optimize survival within competitive ecosystems.

In addition to AI-2 signaling, *Salmonella* responds to autoinducer-3 (AI-3) and host-derived catecholamines through sensor kinase systems such as QseC (Bearson and Bearson, 2008; Zhang et al., 2022; Yang et al., 2026). This form of interkingdom signaling enables bacterial populations to detect physiological changes within the host and modify virulence accordingly. Host stress hormones, including epinephrine and norepinephrine, can activate bacterial signaling pathways that enhance motility, attachment, and invasion-associated gene expression, demonstrating that communication extends beyond microbial populations to include direct interactions between host and pathogen (Kendall and Sperandio, 2016). Such multidirectional signaling highlights the intimate relationship between host physiology and microbial ecology within the intestinal environment.

### 3.2 Biofilm-associated interactions and quorum quenching

Quorum sensing also plays a central role in biofilm development, one of the most important ecological adaptations contributing to *Salmonella* persistence in poultry environments (Rana et al., 2021; Sholpan et al., 2021; Chen et al., 2023; Mi et al., 2024). Biofilms are highly organized multicellular structures in which bacterial cells are embedded within extracellular polymeric substances that facilitate nutrient sharing, stress tolerance, and coordinated gene expression. Within these structures, signaling molecules accumulate at high concentrations, promoting synchronized responses that enhance resistance to disinfectants, desiccation, oxidative stress, and antimicrobial agents (Sholpan et al., 2021; Małaszczuk et al., 2025). The enhanced resilience of biofilm-associated populations partly explains the long-term persistence of *Salmonella* in poultry houses, water systems, and processing facilities despite repeated sanitation procedures.

Recognition of the importance of microbial communication pathways has stimulated interest in quorum quenching strategies that disrupt bacterial communication without directly inhibiting growth. Quorum-sensing inhibitors derived from plants, microorganisms, enzymes, and synthetic compounds can interfere with signaling pathways by degrading autoinducers or blocking receptor activation, thereby reducing biofilm formation and virulence expression while imposing less selective pressure for antimicrobial resistance than conventional antibiotics (Mi et al., 2024; Goswami, 2025). These approaches represent promising alternatives for sustainable control of *Salmonella* through ecological modulation rather than bacterial eradication.

However, several limitations currently restrict the practical application of quorum-quenching approaches. Much of the available evidence is derived from *in vitro* studies, and activity observed under controlled conditions may not be reproduced within complex intestinal microbiota, established multispecies biofilms, or variable poultry production environments. The efficacy of quorum-sensing inhibitors may also depend on compound stability, concentration, delivery, and accessibility to signaling molecules or receptors. Moreover, interference with broadly conserved signaling systems may affect non-target members of the microbiota, potentially producing unintended ecological effects. Importantly, although quorum quenching is generally expected to impose less selective pressure than bactericidal antimicrobials, resistance or reduced susceptibility to quorum-sensing inhibitors can evolve through mechanisms such as altered receptors or increased efflux. Therefore, quorum quenching should presently be considered a promising complementary strategy rather than a stand-alone alternative to established *Salmonella* control measures (Krzyżek, 2019; Mi et al., 2024; Goswami, 2025).

### 3.3 Metabolic cross-feeding and environmental modification

In addition to dedicated signaling, ecological interactions such as metabolic cross-feeding can influence *Salmonella* by modifying the biochemical environment rather than through a specific signal-receptor system. Microbial populations continuously exchange metabolites, including short-chain fatty acids, amino acids, vitamins, and organic acids that function as both nutrients and signaling compounds (Henriques et al., 2020; Portincasa et al., 2022; Zhang et al., 2023; Liu et al., 2025). Such metabolic cross-feeding establishes interdependencies among community members and can indirectly regulate *Salmonella* growth and virulence. Butyrate and propionate produced by anaerobic fermentative bacteria, for example, have been associated with reduced invasion gene expression and enhanced colonization resistance, illustrating how microbial metabolism influences pathogen behavior beyond simple nutrient competition (Maurer et al., 2024; Schultz Marcolla et al., 2025).

Metabolic communication also contributes to niche partitioning within complex microbial communities. Different bacterial populations specialize in distinct metabolic functions while sharing intermediate metabolites, creating cooperative networks that maximize ecosystem efficiency (Taillefumier et al., 2017; Douglas, 2020; Nef et al., 2024). Alterations in these networks may profoundly affect *Salmonella* physiology. Comparative analyses of restrictive and permissive cecal communities demonstrated significant differences in pathways associated with propanediol utilization, vitamin B12 biosynthesis, and lipopolysaccharide modification, resulting in reduced pathogen fitness and suppression of SPI-1 virulence genes within exclusionary microbial communities (Maurer et al., 2024). These findings suggest that microbial communication through metabolism can reshape pathogen physiology without direct antagonistic interactions.

### 3.4 Extracellular vesicle-mediated communication

Recent evidence further indicates that extracellular vesicles and secreted biomolecules contribute to microbial communication within polymicrobial communities (Limoli et al., 2015; Douglas, 2020; Díaz-Garrido et al., 2021; Federica et al., 2025; Kawagishi et al., 2025). Membrane vesicles transport proteins, nucleic acids, enzymes, and signaling molecules between bacterial populations, facilitating coordinated responses to environmental stress while promoting horizontal gene transfer and community adaptation (Zhang L. et al., 2026). Although extracellular vesicle-mediated communication is well documented in several bacterial systems, direct evidence establishing its ecological importance for *Salmonella* within poultry-associated microbial communities remains limited. Therefore, its role in poultry should presently be regarded as a plausible emerging mechanism rather than a firmly established determinant of persistence (Kendall and Sperandio, 2016; Jang et al., 2025).

Environmental modification represents another indirect ecological process rather than a dedicated communication system. In this case, one microbial population alters physicochemical or resource conditions that subsequently influence another organism without requiring signal recognition (Hassani et al., 2018; Geesink et al., 2024; Liu et al., 2024; Srinivasan et al., 2024). Such environmental modification may involve changes in oxygen availability, pH, redox conditions, iron availability, or bile acid composition, all of which can influence *Salmonella* physiology under appropriate conditions. However, the extent to which these processes are directly mediated by resident poultry microbiota and causally contribute to *Salmonella* exclusion varies among mechanisms and remains incompletely established. Depletion of oxygen, alteration of pH, sequestration of iron, or modification of bile acid composition can generate cascading effects throughout microbial communities that ultimately regulate *Salmonella* colonization (Khan et al., 2021). Such indirect interactions may exert greater ecological influence than direct competition because they alter the functional characteristics of the entire ecosystem rather than targeting individual pathogens (Jang et al., 2025). This perspective aligns with emerging ecological theories emphasizing network properties and community interactions over species-specific mechanisms.

### 3.5 Horizontal gene transfer as a related evolutionary process

Horizontal gene transfer (HGT) is conceptually distinct from microbial communication because it involves the acquisition of heritable genetic material rather than signal-mediated regulation of cellular behavior. HGT occurs principally through conjugation, transduction, and transformation, with mobile genetic elements such as plasmids, bacteriophages, transposons, and integrative elements contributing to bacterial genome evolution. In *Salmonella*, these processes can disseminate antimicrobial resistance, virulence, and other adaptive determinants that influence fitness and persistence. Poultry-derived *Salmonella* isolates have been shown to harbor plasmids carrying antimicrobial-resistance and virulence genes with potential for conjugative transfer, demonstrating the relevance of HGT within poultry-associated populations. HGT is therefore considered here as a related evolutionary process that can modify the functional traits of *Salmonella* and surrounding microbial communities, rather than as a microbial communication pathway (Lin et al., 2025).

Importantly, the signaling and ecological mechanisms described above do not operate independently but interact across spatial and functional scales to influence *Salmonella* persistence. Quorum-sensing responses can promote biofilm development, while the biofilm matrix and high local cell density can, in turn, favor the accumulation of signaling molecules and coordinated stress responses (Sholpan et al., 2021; Mi et al., 2024; Małaszczuk et al., 2025). At the same time, metabolic activity within microbial communities modifies nutrient availability and generates metabolites such as butyrate and propionate that can alter *Salmonella* fitness and virulence expression (Maurer et al., 2024; Schultz Marcolla et al., 2025). These metabolic changes occur alongside environmental modifications and host-derived signals, creating local conditions that may either restrict or favor pathogen survival. Extracellular vesicles may provide an additional layer of interaction by transporting proteins, nucleic acids, enzymes, and signaling molecules, although their specific contribution to poultry-associated *Salmonella* ecosystems remains poorly defined. Thus, *Salmonella* persistence is more appropriately viewed as an emergent outcome of interacting signaling, metabolic, biofilm-associated, and ecological processes rather than the consequence of a single communication pathway. However, direct poultry-specific studies simultaneously examining these mechanisms remain limited, and many proposed interactions are currently supported by evidence from individual mechanisms or other microbial systems rather than by integrated experimental studies.

Collectively, dedicated signaling systems and broader ecological interactions, including quorum sensing, interkingdom signaling, metabolic cross-feeding, biofilm-associated interactions, and environmental modification, interact to influence *Salmonella* behavior within poultry environments. These signaling pathways enable pathogens to continuously sense and respond to surrounding microbial communities and host conditions, thereby optimizing survival and transmission. Understanding these communication systems provides the mechanistic foundation for developing ecosystem-based interventions that disrupt pathogenic networks while preserving beneficial microbial functions, offering a promising direction for sustainable control of *Salmonella* in modern poultry production. The principal signaling and ecological mechanisms influencing *Salmonella* behavior are summarized in Table 2, while their functional integration and relationship with horizontal gene transfer are illustrated in Figure 2.

**TABLE 2:** Signaling and ecological mechanisms influencing *Salmonella* colonization, persistence, and virulence.

Mechanism	Signal	Biological consequence
AI-2 quorum sensing	Autoinducers	Virulence regulation
AI-3/QseC	Host hormones	Interkingdom signaling
Metabolic cross-feeding	SCFAs, vitamins	Colonization resistance
Biofilm communication	EPS matrix	Environmental persistence
Extracellular vesicles	Proteins/RNA	Community adaptation
Indirect ecological interactions	Environmental modification	Ecosystem restructuring

**FIGURE 2:**
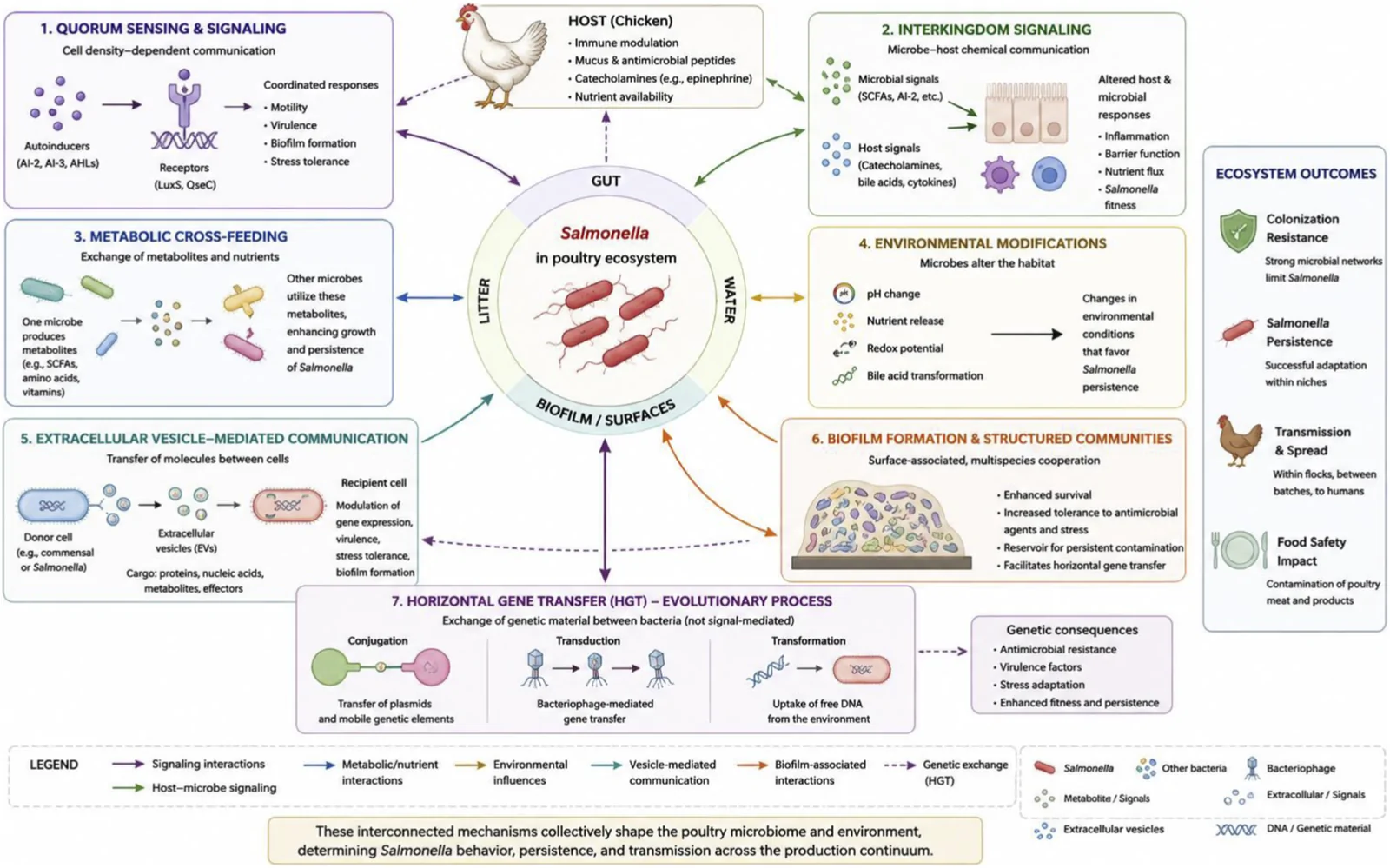
Conceptual integration of signaling, ecological interactions, and horizontal gene transfer influencing *Salmonella* ecology in poultry environments. Dedicated signaling mechanisms include quorum sensing and interkingdom signaling, whereas metabolic cross-feeding, environmental modification, extracellular vesicle-mediated communication, and biofilm-associated interactions influence pathogen behavior through distinct but interconnected processes. Horizontal gene transfer is shown separately as a related evolutionary process involving heritable genetic exchange rather than signal-mediated communication. Together, these mechanisms influence *Salmonella* colonization, persistence, adaptation, and transmission within poultry-associated ecosystems.

## 4 Ecological interactions shaping *Salmonella* colonization and persistence

The ecological success of *Salmonella* in poultry production systems is determined by its ability to establish itself within complex microbial communities rather than by virulence factors alone. Within the intestinal tract and environmental reservoirs, *Salmonella* encounters numerous microorganisms competing for nutrients, attachment sites, and ecological niches while simultaneously participating in cooperative and antagonistic interactions that collectively determine community structure (Khan et al., 2021; Singh and Koo, 2024). Consequently, pathogen colonization is increasingly recognized as an emergent property of ecosystem dynamics, where the balance among microbial populations governs resistance or susceptibility to invasion (Gart et al., 2016; Kogut and Fernandez Miyakawa, 2023; Nickodem et al., 2025).

Colonization resistance is one of the most important ecological functions of a healthy microbial community and represents the natural ability of resident microorganisms to prevent the establishment of invading pathogens (Seekatz et al., 2022; Woelfel et al., 2024; Li and Guo, 2026). Although traditionally attributed to competition for nutrients and epithelial attachment sites, current evidence indicates that colonization resistance arises from multiple interacting mechanisms, including metabolic competition, bacteriocin production, immune modulation, environmental modification, and microbial signaling. The effectiveness of these mechanisms depends on the overall stability and functional diversity of the microbial ecosystem rather than the abundance of individual bacterial taxa (Maurer et al., 2024; Wongkuna et al., 2024).

However, the strength of evidence supporting these ecological mechanisms is not uniform. Poultry studies provide direct experimental support for competitive exclusion, metabolic restriction, and microbiota-mediated suppression of *Salmonella*, whereas evidence for some broader community-level interactions is derived from associations between microbial composition, metabolite profiles, and pathogen abundance (Maurer et al., 2024; Mekonnen et al., 2024; Malli et al., 2025). Moreover, reductions in *Salmonella* following microbiome manipulation cannot necessarily be attributed to microbial diversity alone because community composition, metabolic function, host responses, bird age, diet, and environmental conditions may contribute simultaneously. Therefore, associations between particular microbial communities and reduced *Salmonella* abundance should be distinguished from experimentally demonstrated causal mechanisms.

Competition for limiting nutrients constitutes a major ecological pressure influencing *Salmonella* colonization. Resident microbiota efficiently utilize available carbohydrates, amino acids, micronutrients, and trace elements, reducing ecological opportunities for invading pathogens (Horrocks et al., 2023; Woelfel et al., 2024). However, nutrient competition extends beyond simple resource depletion and includes complex metabolic interactions in which one microbial population modifies substrate availability for another. Cross-feeding networks involving fermentation products, organic acids, and vitamins create biochemical environments that favor specific microbial populations while restricting pathogen expansion (Culp and Goodman, 2023; Park and Mannaa, 2025; Berretta and Schwab, 2026). Although greater microbial diversity has frequently been associated with colonization resistance, diversity alone should not be considered a direct determinant of *Salmonella* exclusion. Rather, resistance appears to depend on the functional composition of the community, including nutrient competition, metabolite production, niche occupation, and the presence of specific antagonistic or metabolically active taxa (Maurer et al., 2024; Wongkuna et al., 2024; Schultz Marcolla et al., 2025).

Short-chain fatty acids (SCFAs), particularly acetate, propionate, and butyrate, represent key metabolic mediators linking microbial ecology with pathogen regulation (Ney et al., 2023; Nireeksha et al., 2025). Produced through fermentation of dietary substrates by commensal anaerobes, SCFAs influence intestinal pH, epithelial barrier integrity, immune function, and bacterial gene expression (Liu et al., 2021; Ali et al., 2022; Aminullah et al., 2026). Increased concentrations of propionate and valerate associated with defined bacterial communities have been correlated with reduced *Salmonella* abundance and improved colonization resistance, suggesting that microbial metabolites function as ecological signals regulating pathogen fitness (Schultz Marcolla et al., 2025). Similarly, alterations in microbial metabolic pathways involving propanediol utilization and vitamin B12 biosynthesis have been associated with suppression of *Salmonella* growth and virulence in exclusionary cecal communities (Maurer et al., 2024).

Competitive exclusion (CE) provides one of the earliest and most extensively investigated microbiota-based approaches for controlling *Salmonella* in poultry (Lee et al., 2023; Peruzzo et al., 2026). Poultry-specific studies provide direct experimental support for this concept. Defined bacterial communities derived from chicken intestinal microbiota have reduced *Salmonella* colonization while improving intestinal health, demonstrating that protective effects can be reproduced using selected microbial consortia (Wongkuna et al., 2024). Similarly, inoculation of broiler chicks with defined microbial communities altered cecal community structure, increased phylogenetic diversity, modified fermentation metabolites, and reduced Enterobacteriaceae and *Salmonella* abundance (Schultz Marcolla et al., 2025). Importantly, these findings do not demonstrate that increased diversity itself causes *Salmonella* exclusion; rather, they support a functional model in which community composition, metabolic activity, nutrient competition, and specific microbial taxa collectively contribute to colonization resistance.

The inclusion of taxa such as *Megamonas hypermegale* altered fermentation patterns and metabolite production while reducing pathogen abundance, suggesting that specific microbial populations may exert disproportionate effects on community function and colonization resistance (Schultz Marcolla et al., 2025). Nevertheless, the protective effects of CE communities are not necessarily attributable to a single taxon or mechanism, and efficacy can vary according to microbial composition, administration strategy, host age, diet, and challenge conditions. Consequently, correlations between enrichment of particular taxa or metabolites and reduced *Salmonella* abundance should not automatically be interpreted as evidence of direct causality. Mechanistic studies using defined communities, targeted microbial manipulation, and functional analyses are therefore needed to identify which community members and metabolic activities are necessary and sufficient for reproducible colonization resistance (Maurer et al., 2024).

Direct antagonism and indirect ecological interactions should be distinguished according to whether the inhibitory factor acts directly on *Salmonella* or modifies the surrounding ecological niche. Direct antagonism includes the production of bacteriocins, antimicrobial peptides, and other inhibitory compounds that act directly on the pathogen (Caballero-Flores et al., 2023; Deng and Wang, 2024; Singh and Koo, 2024). In contrast, indirect ecological interactions alter environmental conditions or resource availability, including oxygen tension, redox conditions, iron availability, and bile acid composition, which can subsequently influence *Salmonella* fitness and colonization. Experimental studies demonstrate that respiratory metabolism and the availability of oxygen or alternative electron acceptors influence *Salmonella* intestinal fitness, including direct experimental investigation in chickens (Barrow et al., 2015). Iron availability also influences *Salmonella* intestinal fitness, and competition for iron has been experimentally demonstrated to restrict *S. Typhimurium* colonization in mouse models (Deriu et al., 2013). Bile and individual bile acids can directly alter *Salmonella* physiology and repress invasion-associated gene expression, although these effects are context-dependent and have been characterized primarily under experimental conditions rather than as a demonstrated microbiota-mediated mechanism of colonization resistance in poultry (Eade et al., 2016). However, evidence that resident poultry microbiota suppress *Salmonella* specifically through microbiota-mediated oxygen depletion, redox modification, iron sequestration, or bile acid alteration remains uneven, and several mechanistic demonstrations derive from mammalian or *in vitro* models rather than poultry (Deng and Wang, 2024). These processes should therefore be regarded as experimentally supported influences on *Salmonella* physiology with varying levels of evidence for their causal contribution to microbiota-mediated colonization resistance in poultry.

Ecological facilitation can also influence pathogen persistence under certain conditions. Cooperative metabolic interactions among microbial populations may inadvertently generate nutrients or environmental conditions that support *Salmonella* survival, particularly following disturbances such as antibiotic treatment or environmental stress (Hockenberry et al., 2021; Grzymajlo, 2022). Experimental microbial communities have demonstrated that facilitative interaction networks evolve over time and that positive feedback loops often precede major shifts in community composition, suggesting that microbial communication can either stabilize or destabilize ecosystem structure depending on network architecture (Fujita et al., 2023). Understanding these feedback mechanisms may help predict ecological transitions associated with pathogen emergence.

The host immune system forms an integral component of microbial ecological networks and contributes significantly to colonization resistance (Li and Guo, 2026). Resident microbiota stimulate epithelial maturation, mucus production, antimicrobial peptide secretion, and development of mucosal immune responses that collectively restrict pathogen invasion (Buffie and Pamer, 2013; Belkaid and Hand, 2014; Pereira et al., 2022). Conversely, *Salmonella* exploits inflammatory responses to generate ecological advantages by altering nutrient availability and suppressing competing anaerobic populations (Robinson et al., 2022). During persistent colonization, expansion of regulatory T cells and immunometabolic reprogramming shift the intestinal environment toward disease tolerance, enabling long-term asymptomatic carriage while maintaining ecological equilibrium between host and pathogen (Kogut and Fernandez Miyakawa, 2023). These findings demonstrate that immune responses and microbial ecology are tightly interconnected components of the same communication network.

Environmental ecosystems also contribute substantially to *Salmonella* persistence through ecological interactions occurring outside the host. Reused poultry litter contains diverse microbial communities whose composition is influenced by moisture, pH, ammonia concentration, and management practices (Valeris-Chacin et al., 2021; Gontar et al., 2022). Certain bacterial taxa within reused litter exhibit negative correlations with *Salmonella* abundance, while *Bacillus subtilis* consortia isolated from litter have demonstrated inhibitory activity against *Salmonella Heidelberg*, supporting the concept that environmental microbiomes can naturally suppress pathogen survival through ecological competition (Bucher et al., 2020). Similar interactions occur within waterline biofilms and farm environments, where multispecies communities create structured habitats that facilitate microbial communication and influence pathogen persistence (Ogundipe et al., 2025).

Microbiome modulation strategies provide additional evidence for the importance of ecological interactions in pathogen control. Supplementation with galacto-oligosaccharides and attenuated *Salmonella* strains accelerated pathogen clearance by reshaping gut microbial communities rather than directly eliminating the pathogen, illustrating that restoration of ecological balance can enhance host resistance through community-wide effects (Azcarate-Peril et al., 2018). Likewise, commensal microorganisms such as *Lactobacillus paracasei* have demonstrated the capacity to suppress *Salmonella Pullorum* while simultaneously improving microbial diversity, highlighting the therapeutic potential of ecosystem restoration (Zhu et al., 2024).

Collectively, these findings indicate that *Salmonella* colonization and persistence are governed by a complex network of competitive, facilitative, metabolic, and immune-mediated interactions operating across multiple ecological scales. These findings emphasize that pathogen establishment depends on ecosystem-level interactions that collectively determine colonization success. Future intervention strategies should therefore focus on strengthening ecosystem resilience and restoring beneficial microbial interactions rather than solely targeting pathogen elimination, thereby promoting long-term stability and sustainable control of *Salmonella* in poultry production systems.

## 5 Manipulating microbial communication networks for sustainable *Salmonella* control

Traditional approaches for controlling *Salmonella* in poultry have relied primarily on direct pathogen elimination through antibiotics, chemical disinfectants, and vaccination. Although these interventions have contributed to reductions in disease prevalence, their long-term effectiveness is often compromised by antimicrobial resistance, biofilm formation, ecological disruption, and rapid recolonization from environmental reservoirs (Velhner et al., 2018; Hameed et al., 2024; Małaszczuk et al., 2025). Increasing recognition that *Salmonella* functions within complex microbial communication networks has shifted attention toward ecosystem-based interventions that strengthen microbial resilience and suppress pathogen establishment by modifying ecological interactions rather than directly targeting the pathogen.

CE remains one of the most successful examples of ecological manipulation in poultry production. The original concept of administering intestinal contents from healthy adult birds has evolved into the development of defined microbial consortia designed to restore colonization resistance through coordinated microbial interactions (Zaytsoff et al., 2022; Zhang et al., 2025). Rather than acting through a single mechanism, CE cultures simultaneously compete for nutrients and attachment sites, produce antimicrobial compounds, alter metabolite profiles, and modulate host immunity, thereby creating an ecosystem that is unfavorable for *Salmonella* colonization (Maurer et al., 2024; Wongkuna et al., 2024). This multifactorial mode of action explains the greater durability of ecological interventions compared with conventional antimicrobial treatments.

Recent studies have demonstrated that defined bacterial communities can restructure the cecal ecosystem and significantly reduce *Salmonella* abundance while improving microbial diversity and intestinal health (Pedroso et al., 2021). Inclusion of metabolically active taxa such as *M. hypermegale* increased concentrations of beneficial fermentation products, including propionate and valerate, which were associated with enhanced colonization resistance and suppression of Enterobacteriaceae (Schultz Marcolla et al., 2025). These findings support the concept that rational design of microbial consortia based on ecological function rather than taxonomy alone may provide more predictable and reproducible pathogen control strategies.

Prebiotics and synbiotics represent complementary approaches for manipulating microbial communication networks by selectively stimulating beneficial microbial populations and their metabolic activities (Kezer et al., 2025). Supplementation with galacto-oligosaccharides has been shown to modify gut microbial composition and accelerate clearance of *Salmonella* infections, particularly when combined with attenuated *Salmonella* strains that reshape microbial interactions within the intestinal ecosystem (Azcarate-Peril et al., 2018; Richards et al., 2024). By promoting the expansion of fermentative bacteria and increasing production of short-chain fatty acids, prebiotics strengthen colonization resistance while enhancing epithelial integrity and immune function, illustrating how nutritional interventions can indirectly regulate pathogen ecology.

Probiotic microorganisms also exert protective effects through multiple communication pathways. Species of *Lactobacillus*, *Bacillus*, *Bifidobacterium*, and other commensal bacteria produce bacteriocins, organic acids, and signaling molecules that inhibit pathogen growth while simultaneously influencing host immune responses and microbial community structure (Naeem and Bourassa, 2024; 2025). For example, *L. paracasei* effectively inhibited *Salmonella Pullorum* proliferation while restoring microbial diversity in infected chickens, demonstrating that probiotic efficacy extends beyond direct antagonism to include ecosystem restoration and communication-mediated regulation of microbial populations (Zhu et al., 2024). Similarly, *Bacillus subtilis* strains isolated from reused poultry litter reduced *Salmonella Heidelberg* growth, highlighting the potential of environmentally adapted microorganisms as biological control agents (Bucher et al., 2020).

Disruption of quorum-sensing pathways has emerged as another promising strategy for ecological control of *Salmonella* (Zhang et al., 2022). Because quorum sensing regulates biofilm formation, motility, stress tolerance, and virulence expression, inhibition of bacterial communication can reduce pathogen fitness without exerting the strong selective pressure associated with bactericidal compounds. Plant-derived phytochemicals, microbial enzymes, synthetic analogs, and naturally occurring quorum-quenching compounds have demonstrated the capacity to interfere with autoinducer synthesis or receptor recognition, thereby suppressing coordinated pathogenic behaviors (Mi et al., 2024; Goswami, 2025). Unlike bactericidal antibiotics, quorum-sensing inhibitors primarily target bacterial behavior rather than viability and may therefore impose different or potentially lower selective pressures; however, the possibility of adaptation or resistance to quorum-sensing inhibition cannot be excluded (Krzyżek, 2019).

Targeting biofilm-associated communication networks represents another attractive intervention strategy. Biofilms provide structured environments in which microbial populations exchange nutrients and signaling molecules while gaining protection from disinfectants and environmental stress (Zhao et al., 2023). Enzymatic degradation of extracellular polymeric substances, bacteriophage therapy, antimicrobial peptides, and biofilm-resistant surface technologies have shown promise for disrupting biofilm integrity and reducing environmental persistence of *Salmonella* (Goswami, 2025; Małaszczuk et al., 2025). Combining biofilm disruption with ecological restoration strategies may provide synergistic benefits by simultaneously eliminating persistent reservoirs and promoting recolonization by beneficial microbial communities.

Bacteriophage therapy offers an additional ecosystem-based approach that selectively targets *Salmonella* while preserving much of the surrounding microbiota (Hu et al., 2018; Muneeb et al., 2025). Unlike broad-spectrum antimicrobials, phages exert narrow host specificity and therefore minimize collateral disruption of microbial ecosystems that contribute to colonization resistance. Integration of phage therapy with probiotics or CE cultures has been proposed as a strategy to reduce pathogen abundance while facilitating restoration of beneficial microbial networks, thereby enhancing long-term ecosystem stability (Małaszczuk et al., 2025; Nickodem et al., 2025).

Emerging evidence suggests that future microbiome engineering should focus on keystone taxa rather than overall microbial diversity. Keystone microorganisms exert disproportionate influence on ecosystem function through metabolic interactions and communication networks that regulate community assembly and resilience (Tudela et al., 2021; Espíndola-Hernández et al., 2026). Identification and targeted enrichment of such organisms may allow precise manipulation of microbial ecosystems to suppress *Salmonella* while maintaining desirable production characteristics. Ecological network analyses and multi-omics approaches are increasingly facilitating the identification of these functionally important taxa and their associated interaction pathways (Fujita et al., 2023; Jang et al., 2025).

Artificial intelligence, systems biology, and ecological modeling are expected to further accelerate the development of precision microbiome interventions. Integration of metagenomic, metatranscriptomic, metabolomic, and ecological network datasets may enable prediction of microbial community responses to dietary, environmental, or management interventions before implementation in commercial settings. Such predictive models could identify microbial communication pathways critical for ecosystem resilience and guide rational design of microbiome-targeted strategies that maximize colonization resistance while minimizing production losses (Jang et al., 2025; Nickodem et al., 2025).

From a One Health perspective, ecosystem-based interventions offer significant advantages over pathogen-directed approaches by reducing antimicrobial use, limiting selection for antimicrobial resistance, and enhancing overall microbial stability throughout the poultry production chain. Rather than attempting complete eradication of *Salmonella*, which is often impractical in complex agricultural environments, strengthening microbial communication networks and ecosystem resilience may provide a more sustainable means of maintaining pathogen populations below thresholds associated with disease transmission and food safety risks (Hameed et al., 2024; Wigley, 2024).

Overall, manipulation of microbial communication networks represents a paradigm shift in *Salmonella* control, moving from direct antimicrobial intervention toward ecological engineering of the poultry microbiome. CE cultures, defined microbial consortia, prebiotics, probiotics, quorum-sensing inhibitors, biofilm-disrupting technologies, bacteriophages, and precision microbiome engineering collectively demonstrate that restoration of ecosystem function can provide durable suppression of *Salmonella*. Continued integration of microbial ecology with systems biology and network analysis will likely underpin the next-generation of sustainable intervention strategies for improving poultry health and food safety. The major ecosystem-based intervention strategies targeting microbial communication are summarized in Figure 3, and current and emerging microbiome-based intervention strategies are compared in Table 3.

**FIGURE 3:**
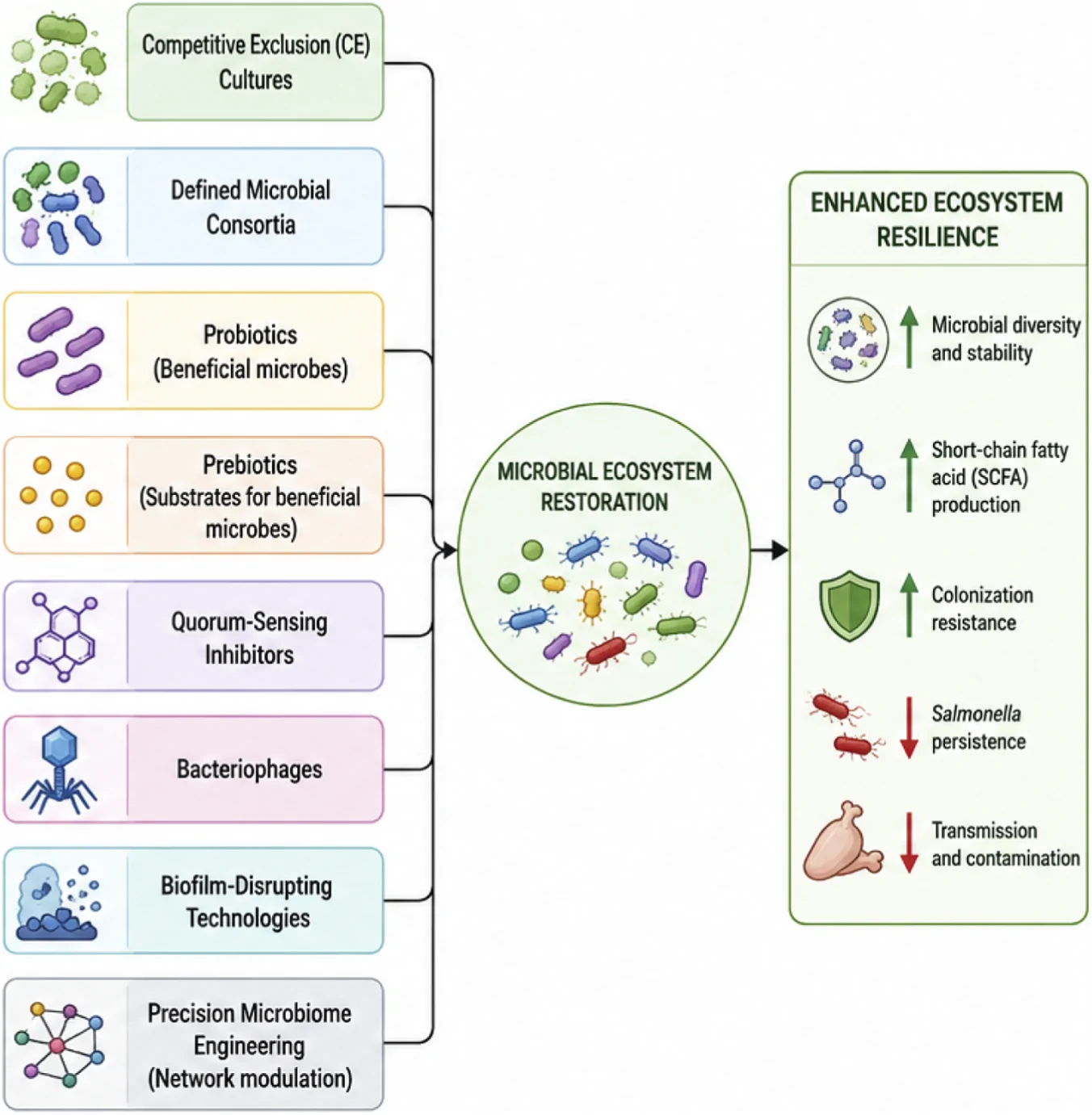
Ecosystem-based approaches for manipulating microbial communication networks to suppress *Salmonella* in poultry. Illustration of competitive exclusion cultures, defined microbial consortia, probiotics, prebiotics, quorum-sensing inhibitors, bacteriophages, biofilm-disrupting technologies, and precision microbiome engineering converging to enhance ecosystem resilience, colonization resistance, and food safety.

**TABLE 3:** Microbial communication-based intervention strategies for sustainable *Salmonella* control in poultry.

Strategy	Primary target	Main mechanism	Future potential
Competitive exclusion	Gut microbiota	Community restoration	High
Defined consortia	Keystone taxa	Ecological engineering	High
Probiotics	Beneficial microbes	Competitive inhibition	High
Prebiotics	Microbial metabolism	SCFA production	Moderate-high
Quorum-sensing inhibitors	Communication pathways	Virulence suppression	High
Bacteriophages	*Salmonella*	Targeted lysis	Moderate-high
Biofilm disruption	Environmental reservoirs	Matrix degradation	High
Precision microbiome engineering	Whole ecosystem	Network optimization	Very high

## 6 Future perspectives

Recent advances in microbial ecology have fundamentally changed our understanding of *Salmonella* colonization in poultry by demonstrating that pathogen success is governed by complex communication networks rather than individual virulence determinants alone. Future research should therefore move beyond descriptive analyses of microbial composition toward functional characterization of communication networks that regulate pathogen behavior under commercial production conditions.

A major challenge remains the integration of multiple layers of biological information into a unified ecological framework. Most current studies rely on 16S rRNA sequencing, which provides valuable taxonomic information but limited insight into functional interactions among microbial populations. The application of metagenomics, metatranscriptomics, metaproteomics, and metabolomics offers opportunities to identify signaling pathways, metabolic cross-feeding networks, and regulatory mechanisms that underpin ecosystem stability and *Salmonella* persistence. Integrating these complementary approaches will facilitate the identification of functionally important microbial interactions that cannot be detected through compositional analyses alone.

Ecological network analysis is likely to become an increasingly valuable tool for identifying keystone taxa that exert disproportionate influence on microbial community structure and pathogen exclusion. Rather than targeting highly abundant microorganisms, future interventions may focus on strategically manipulating these functionally important populations to restore ecological balance and strengthen colonization resistance. Identification of microbial hubs and interaction bottlenecks may also reveal novel therapeutic targets capable of suppressing *Salmonella* through ecosystem restructuring rather than direct antimicrobial activity.

Another important research priority is understanding spatial organization within microbial communities. Most investigations evaluate homogenized intestinal or environmental samples, overlooking microscale heterogeneity that determines microbial communication and metabolic exchange. Advanced imaging technologies, spatial transcriptomics, and single-cell sequencing could provide unprecedented insights into how *Salmonella* interacts with neighboring microorganisms within biofilms, intestinal mucus layers, and litter microhabitats. Such spatially resolved analyses may reveal localized ecological interactions that are obscured by bulk community analyses.

The role of environmental microbiomes also deserves greater attention. Hatcheries, reused litter, waterline biofilms, transport equipment, and processing environments are increasingly recognized as interconnected microbial reservoirs that contribute to pathogen persistence and transmission across production cycles. Future studies should therefore adopt a farm-to-processing systems approach, recognizing that microbial communication extends beyond the gastrointestinal tract to encompass the entire poultry production ecosystem. Longitudinal studies tracking microbial network dynamics across successive flocks would substantially improve understanding of pathogen ecology and environmental resilience.

Precision microbiome engineering represents one of the most promising future directions for sustainable *Salmonella* control. Instead of introducing individual probiotic strains, rationally designed microbial consortia based on ecological function may provide greater stability and reproducibility under commercial conditions. Recent studies demonstrating successful suppression of *Salmonella* using defined bacterial communities illustrate the feasibility of engineering microbial ecosystems that enhance colonization resistance through coordinated metabolic and signaling interactions. Incorporating ecological principles into probiotic development may therefore overcome many limitations associated with conventional single-strain interventions.

Similarly, quorum-sensing inhibition and ecological modulation offer attractive alternatives to traditional antimicrobial approaches. By disrupting bacterial communication and biofilm development without directly inhibiting bacterial growth, quorum quenching strategies may reduce selective pressure for antimicrobial resistance while preserving beneficial microbial communities. Integration of quorum-sensing inhibitors with CE cultures, prebiotics, bacteriophages, or microbiome engineering could provide synergistic approaches for long-term pathogen suppression.

Artificial intelligence and computational ecology are expected to play an increasingly important role in interpreting the complexity of microbial communication networks. Machine learning algorithms capable of integrating multi-omics datasets with environmental and production variables may enable the prediction of ecosystem responses to dietary modifications, management practices, or intervention strategies before implementation in commercial flocks. Such predictive ecological models could support precision poultry management by identifying conditions that favor microbial resilience while minimizing opportunities for *Salmonella* colonization.

From a One Health perspective, ecosystem-based management strategies align closely with global efforts to reduce antimicrobial use while improving animal health and food safety. Strengthening beneficial microbial communication networks may decrease pathogen carriage, limit environmental contamination, reduce dissemination through the food chain, and minimize the emergence of antimicrobial resistance, thereby benefiting both animal and public health. Such approaches recognize that sustainable disease control depends on maintaining ecological balance rather than pursuing complete pathogen eradication.

In conclusion, *Salmonella* should no longer be regarded as an isolated pathogen but as an active participant within highly interconnected microbial communication networks that span the intestinal tract and the broader poultry production environment. Quorum sensing, metabolic cross-feeding, indirect ecological interactions, host-derived signals, and environmental biofilms collectively regulate pathogen colonization, persistence, and transmission. Future control strategies should therefore prioritize restoration and engineering of microbial ecosystem function rather than reliance on pathogen-directed interventions alone. Continued integration of microbial ecology, systems biology, network analysis, and precision microbiome engineering is expected to transform the prevention and control of *Salmonella* and support the development of more sustainable poultry production systems. The major communication mechanisms and intervention strategies discussed throughout this review collectively support a transition from pathogen-centered control toward ecosystem-based management of *Salmonella* in poultry production.

## 7 Conclusion

Evidence synthesized in this review demonstrates that *Salmonella* ecology in poultry is governed by complex microbial communication networks rather than pathogen virulence alone, highlighting the central role of ecosystem-level interactions in colonization resistance and persistence. Communication through quorum sensing, metabolic cross-feeding, indirect ecological interactions, biofilm formation, and host-derived signaling molecules collectively regulates pathogen colonization, persistence, and transmission across the gastrointestinal tract and environmental reservoirs. These findings challenge traditional pathogen-centered paradigms and support a broader ecosystem-based perspective in which microbial community function determines colonization resistance and ecosystem resilience. Evidence reviewed here indicates that restoration or manipulation of beneficial microbial networks through CE cultures, defined microbial consortia, prebiotics, probiotics, quorum-sensing inhibitors, and microbiome engineering can suppress *Salmonella* more sustainably than approaches relying solely on antimicrobial interventions. Advances in multi-omics technologies, ecological network analysis, and computational modeling are expected to accelerate the identification of keystone taxa and critical signaling pathways that maintain microbial stability. Future intervention strategies should therefore prioritize preservation and engineering of microbial ecosystem function to enhance poultry health and reduce foodborne transmission. Adopting this systems-level understanding of microbial communication networks offers a promising pathway toward precision microbiome management and more effective, sustainable control of *Salmonella* within modern poultry production systems.
